# Scene analysis in the natural environment

**DOI:** 10.3389/fpsyg.2014.00199

**Published:** 2014-04-01

**Authors:** Michael S. Lewicki, Bruno A. Olshausen, Annemarie Surlykke, Cynthia F. Moss

**Affiliations:** ^1^Department of Electrical Engineering and Computer Science, Case Western Reserve UniversityCleveland, OH, USA; ^2^Helen Wills Neuroscience Institute, School of Optometry, Redwood Center for Theoretical Neuroscience, University of California at BerkeleyBerkeley, CA, USA; ^3^Department of Biology, University of Southern DenmarkOdense, Denmark; ^4^Department of Psychology and Institute for Systems Research, University of MarylandCollege Park, MD, USA

**Keywords:** active perception, auditory streaming, echolocation, vision, electroreception, scene analysis, top-down processes, neuroethology

## Abstract

The problem of scene analysis has been studied in a number of different fields over the past decades. These studies have led to important insights into problems of scene analysis, but not all of these insights are widely appreciated, and there remain critical shortcomings in current approaches that hinder further progress. Here we take the view that scene analysis is a universal problem solved by all animals, and that we can gain new insight by studying the problems that animals face in complex natural environments. In particular, the jumping spider, songbird, echolocating bat, and electric fish, all exhibit behaviors that require robust solutions to scene analysis problems encountered in the natural environment. By examining the behaviors of these seemingly disparate animals, we emerge with a framework for studying scene analysis comprising four essential properties: (1) the ability to solve ill-posed problems, (2) the ability to integrate and store information across time and modality, (3) efficient recovery and representation of 3D scene structure, and (4) the use of optimal motor actions for acquiring information to progress toward behavioral goals.

## INTRODUCTION

In recent decades, research on scene analysis has advanced in many different fields. Perceptual studies have characterized the many cues that contribute to scene analysis capabilities. Computational approaches have made great strides in developing algorithms for processing real-world scenes. Animal behavior and neurobiological studies have investigated animal capabilities and neural representations of stimulus features. In spite of these advances, we believe there remain fundamental limitations in many of the ways scene analysis is defined and studied, and that these will continue to impede research progress until these shortcomings are more widely recognized and new approaches are devised to overcome them. The purpose of this article is to identify these shortcomings and to propose a framework for studying scene analysis that embraces the complex problems that animals need to solve in the natural environment.

A major limitation we see in current approaches is that they do not acknowledge or address the complexity of the problems that need be solved. Experiments based on simplistic, reflexive models of animal behavior, or with the implicit assumption of simple feature detection schemes, have little chance of providing insight into the mechanisms of scene analysis in complex natural settings. An additional limitation lies with the extensive use of “idealized” stimuli and stripped down tasks that yield results which are often difficult to generalize to more ecologically relevant stimuli and behaviors. For example, scene analysis experiments designed around auditory tone bursts are of limited value in helping us to understand how complex acoustic patterns such as speech are separated from noisy acoustic backgrounds. Visual grouping experiments using bar-like stimuli are a far cry from the situation a predator faces in detecting and tracking prey in a complex visual environment. At the same time, computational approaches in the engineering and computer science community, although often applied to natural scenes, have provided only limited insight into scene perception in humans and other animals. The disconnect here is due to the fact that tasks are chosen according to certain technological goals that are often motivated by industrial applications (e.g., image search) where the computational goals are different from those in more ecologically relevant settings. In the neuroscience community, studies of animal behavior and physiology have focused largely on specific stimulus features or assume feedforward processing pipelines that do not address the more complex set of problems required for extraction of these stimulus features in natural scenes.

Here we argue for a view of scene analysis that is broader, more ecological, and which encompasses the diverse set of problems faced by animals. We believe the study of animals is essential for advancing our understanding of scene analysis because, having evolved in complex environments, they have developed a wide range of robust solutions to perceptual problems that make optimal tradeoffs between performance and resource constraints. This premise is important, because it provides a means to develop testable theories and predictive models that do not just describe a set of phenomena, but are based on optimal solutions to well-defined computational goals. Our perspective is similar to that advocated by Marr (1982): scene analysis is fundamentally an information processing task and encompasses a complex set of interrelated problems. What those problems are, however, remain poorly understood. Therefore one of the primary research objectives must be to identify the problems that need to be solved.

## CURRENT APPROACHES TO SCENE ANALYSIS

Here, we examine some of the current perceptual and computational approaches to studying scene analysis. We highlight concepts in these areas that are fundamental to scene analysis and that can inform future experimental and computational approaches. We also discuss the limitations that make current concepts inadequate for understanding scene analysis in the natural environment. Our discussion is guided by two fundamental questions: how do these approaches inform us about the problems that humans and other animals actually need to solve? How do they inform us about the computations required for the analysis of natural scenes?

### PERCEPTUAL STUDIES OF SCENE ANALYSIS

#### Grouping and selective attention

Many perceptual approaches to scene analysis have their roots in the Gestalt studies of visual grouping and related problems of selective attention. These cast scene analysis as a problem of perceptual organization where different elements of the image must be appropriately grouped or “parsed,” e.g., into foreground vs. background or local features belonging to the same contour or surface (Palmer, 1999). Likewise in audition, scene analysis is commonly viewed as the problem of organizing the features in the input into different auditory streams (Bregman, 1990).

While these lines of research have led to the discovery of a wealth of intriguing phenomena, grouping by itself does not yield a representation of objects or 3D scene structure that is adequate to guide purposeful actions. Although it seems reasonable that it may make the problem easier, this assumption is rarely tested in practice. The main problem we face in scene analysis is the *interpretation* of sensory information. We see the figure in the well-known Dalmatian dog image not by determining which blob goes with which other, or by separating foreground from background, but by arriving at an interpretation of the relation among 2D blobs that is consistent with a 3D scene – i.e., it not just the dog, but other aspects of the scene such as the outlines of sidewalk, the shadow cast by an adjacent tree, that are all perceived at once (Yuille and Kersten, 2006). Similar arguments apply to visual feature or region segmentation strategies (e.g., Shi and Malik, 2000). These only give the illusion of solving the problem, because we as observers can look at the result and interpret it. But who is looking at such representations inside the brain? How does the ability to group texture regions or outline the bounding contour of an object within a 2D image translate into directing action toward a point in space? How does it help you navigate a complex 3D scene? It is questionable whether currently studied subproblems of perceptual organization and grouping bring us any closer to understanding the structure of the scene or the mechanisms of its analysis.

Another limitation in many approaches to grouping and attention, and experimental stimuli in general, is that they presume a set of artificial features that do not capture the complexity of natural scenes, e.g., fields of oriented bars or, in audition, sequences of tone pips (Bergen and Julesz, 1983; Bregman, 1990). What most models fail to address is that attending to a feature or grouping a region of the scene is rarely sufficient to solve a perceptual problem, because the desired information (such as object or surface structure) is entangled in complex ways with other structures in the scene, e.g., occluding surfaces, shadows, and the visual (or auditory) background. How does the ability to group tone sequences translate into our ability to perceive speech and other sounds in natural soundscapes? In natural scenes, it is not obvious what the important features are or how to extract them, although more recent work has begun to explore the perceptual organization of contours in natural scenes (Geisler et al., 2001; Elder and Goldberg, 2002; McDermott, 2004), and the role of the context of the natural visual scene itself (Oliva and Torralba, 2007).

Biological systems have evolved in the natural environment where the structure of sensory signals is highly complex. The use of simplified artificial stimuli and tasks are thus testing the system far outside the domain in which it has evolved to operate. The problem with this approach is that the system we seek to characterize is highly non-linear, but unlike linear systems there is no universal way to characterize such systems in terms of a reduced set of functions. The question we need to be asking is, how can we preserve the ecological complexity and relevance of the input and task while still introducing informative experimental manipulations? And for the experimental simplifications and idealizations we choose, can we show that the results generalize to more natural settings?

#### Spatial perception

Animals must act in a 3D world, so the recovery of 3D spatial information sufficient to guide behavior is a fundamental problem in scene analysis. The numerous cues that contribute to 3D spatial perception, such as depth, shape, and spatial layout have been well-studied (Harris and Jenkin, 2011), however most are studied in isolation or in “idealized” settings that bypass more complex processes of scene analysis (see, e.g., Cutting and Vishton, 1995). In natural images, local cues such as disparity or motion are usually highly ambiguous and difficult to estimate reliably. Moreover, many widely studied models are carried out almost exclusively in terms of artificial features embedded in a flat, 2D scene, and it is not clear how these results inform us about scene analysis in the realm of complex 3D scenes typical of sensory experience. Spatial audition is an equally important aspect of scene analysis for both humans and animals, but we have only a limited understanding of how spatial auditory cues such as timing and intensity differences could be extracted from complex acoustic environments (see, e.g., Blauert, 1997; Neuhoff, 2004).

The extraction of low-level features such as disparity or inter-aural timing differences is only the beginning of a complex inference process in scene analysis. A more fundamental issue is the question of what types of spatial information animals need to derive from the scene and how these are represented and integrated with other information sources. Visual cues of spatial layout, such as disparity or motion parallax, are retinocentric and cannot directly drive action without accounting for the movements and positions of the eyes, head, and body (Melcher, 2011). It is often assumed – either implicitly or explicitly – that simply having a representation of the depth of each point in a scene provides a sufficient representation of 3D space. But as Nakayama et al. (1995) have noted, this is not necessarily the case:

Because we have a two-dimensional retina and because we live in a three-dimensional world, many have seen the problem of space perception as the recovery of the third dimension…. Yet there are reasons to think that [Euclidean geometry] is not the manner in which spatial distance is encoded in the visual system. Perceptual psychologist Gibson (1966) argues that space is not perceived in this way but in terms of the surfaces that fill space. The most important and ecologically relevant surface is the ground plane. In Gibson’s view, Euclidian distances between arbitrary points in three-dimensional space are not biologically relevant (see also Nakayama, 1994). We see our world in terms of surfaces and plan our actions accordingly.

Currently, we have a very limited understanding of how surfaces might be computed and represented or to what extent this constitutes an adequate representation of the natural scene.

#### Active perception

Scene analysis can also be viewed as an active process that gathers information about the scene (Ballard, 1991), often in a task-driven manner. This is in contrast to the more standard, passive view that overlooks the contribution of goal-directed action. Hypotheses about the systems goals are essential for gaining insight into underlying computational principles. Active perception models lend themselves to more clearly defined goals, because information is gathered for the task at hand, such as in gaze control, visual search, guidance of limb movement, or locomotion over the immediate terrain (Gibson, 1958; Henderson and Hollingworth, 1999; Lappe et al., 1999; Land and Tatler, 2009; Lederman and Klatzky, 2009; Wolbers and Hegarty, 2010). These studies move closer to ecological relevance, but identifying what drives the acquisition of specific information under natural conditions, and how information is integrated across saccades or probing actions to appropriately guide action, remain open problems.

### COMPUTATIONAL APPROACHES TO SCENE ANALYSIS

Computational approaches to problems of scene analysis began decades ago – Gibson first published his ideas on the process of visual scene analysis in the 1950s (Gibson, 1950, 1958); the cocktail party problem was also first described and studied around the same time by Cherry (1953), when the first speech recognition systems were being built at Bell Labs (Davis et al., 1952). Yet, even today many aspects of scene analysis are still open problems in machine vision and speech processing. Why has it been so hard? Problems can be hard because the right way to approach them is not understood or hard in the sense of computational complexity. Scene analysis is hard for both reasons. Nearly 30 years after these early investigations, Marr (1982) noted that

…in the 1960s almost no one realized that machine vision was difficult…the idea that extracting edges and lines from images might be at all difficult simply did not occur to those who had not tried to do it. It turned out to be an elusive problem. Edges that are of critical importance from a three-dimensional point of view often cannot be found at all by looking at the intensity changes in an image. Any kind of textured image gives a multitude of noisy edge segments; variations in reflectance and illumination cause no end of trouble; and even if an edge has a clear existence at one point, it is as likely as not to fade out quite soon, appearing only in patches along its length in the image.

Evidently, there is a vast gulf between our introspective notions of how we perceive scenes and our realization of what is actually needed to accomplish this. Computational models thus force us to be grounded in our reasoning by testing our assumptions about the types of representations needed for solving a task, and exposing what works and what does not.

#### Ill-posed problems

A formal mathematical reason scene perception is a difficult computational problem is that it is *ill-posed* (Poggio and Koch, 1985; Pizlo, 2001; McDermott, 2009), meaning that there is not enough sensory data available to arrive at a unique solution, and often there are a very large number of possible solutions. Ambiguity in the raw sensory input can only be resolved using *a priori* knowledge about scene structure (Pizlo, 2001; Kersten and Yuille, 2003; Yuille and Kersten, 2006).

An early paradigm for demonstrating the importance of structural knowledge in visual scene analysis was the “blocks world” where the objective is to parse or segment the scene by grouping local 2D edges and junctions into separate (3D) structures (Roberts, 1965; Waltz, 1975; for a more recent perspective, see Frisby and Stone, 2010). Because the role of a local feature is ambiguous and there are a combinatorial number of possible groupings, the problem is not trivial. Although this is a highly reduced problem (and therefore artificial), one of the key insights from this research was that the ambiguity in “bottom-up” information can be resolved by using “top-down” knowledge of structural relationships and optimization algorithms to find the best solutions. More sophisticated approaches can, for example, recover object shapes or rectangular room layouts from photographs (Hoiem and Savarese, 2011).

Some of the most successful approaches to recovering 3D structure use prior knowledge regarding the geometry of corresponding points in the left and right images (or more generally multiple images; Hartley and Zisserman, 2004). These methods mainly recover the Euclidean coordinates of points in the scene, which is of limited relevance to biology, but the underlying mathematics provides a fundamental statement of what information is required, such as inference of the observer’s position and motion in addition to the scene structure. Recovering more complex shapes and surfaces remains an area of active research, but some recent model-based approaches can accurately infer complex 3D shapes such as faces or animals from real images (Blanz and Vetter, 2003; Cashman and Fitzgibbon, 2013).

In the auditory domain, successful scene analysis approaches also make extensive use of statistical inference methods to solve ill-posed problems (Rabiner and Juang, 1993; Gold et al., 2011). The early history of speech recognition was focused largely on feature detection, which is ill-posed due to both the complexity of speech and the presence of other interfering sounds and background noise. The use of hidden Markov models, which integrate temporal context, and statistical learning and inference methods allowed for much more accurate recognition even though the low-level feature representations remained crude and ambiguous. The best systems have proved to be those with the best prior models (Darwin, 2008). Recent speech systems became the first to surpass human performance in a specialized recognition task involving simultaneous masking speech (Cooke et al., 2010). Inference in these models is hierarchical: each level of features, phonemes, words, and sentences tries to deduce the most probable sequence of uncertain elements using both *a priori* knowledge (such as the voice, vocabulary, and grammar) and ongoing contextual information.

Ill-posed inference problems can also be approached sequentially where information is actively gathered, as in active perception. In robotics, a well-studied problem domain is simultaneous localization and mapping (SLAM; Thrun et al., 2005). Here, a robot must use its sensors (typically distance sensors like sonar or laser scanners) to both determine its location in the environment and map out the environment itself. The problem is ill-posed because both the initial position of the robot and the structure of the scene are unknown, and (due to noise) neither the sensors nor the actuators provide precise information about distance or movements. In spite of these challenges, probabilistic approaches have been successful in real-world domains by using statistical inference techniques to build up an accurate representation of the scene from multiple samples and intelligently probe the scene to resolve ambiguity.

Computational approaches to scene analysis inference problems have been successful when they have good prior models of how scenes are generated, which allows accurate interpretation of what would otherwise be highly ambiguous local features. Could they provide insight into biological systems? So far, we would argue they have not. One problem is that these algorithms have focused only on the computational problem and have not been formulated in a way that makes it possible to draw correspondences with neural systems. Another problem is ecological relevance: the choice of problems was not motivated by trying to understand animal behavior, but rather by specific technological goals. Often these problems are defined in narrow settings and are highly simplified to make them tractable (e.g., the blocks world), whereas biology must employ solutions that work in the full complexity of the natural environment. This type of robustness has remained elusive for the majority of computational approaches.

#### Segmentation and grouping

Computationally, grouping can be viewed either as a problem of grouping the correct features or of finding the correct segmentation. For sounds, the problem is to separate mixed sound sources or group features of a single source, as in auditory streaming. This is a difficult problem because in general there are a combinatorial number of groupings. Nevertheless there have been significant advances in developing computational algorithms to find an optimal partitioning of a complex scene from low-level features (Shi and Malik, 2000; Tu and Zhu, 2002; Martin et al., 2004). Most approaches, however, yield a segmentation in terms of the 2D image, which does not reliably provide information about the scene *per se*. For speech and audio, computational auditory scene analysis models based on auditory grouping cues have improved recognition (Cooke et al., 2010), although doing so for natural sounds in real acoustic environments remains a challenge. Techniques such as blind source separation (Bell and Sejnowski, 1995; Hyvarinen et al., 2004) can de-mix arbitrary sounds but only under very restrictive assumptions. None of these approaches, however, match the robustness of biological systems.

Segmentation is often considered to be a pre-requisite for recognition (discussed in more detail below), but that need not be the case. An alternative approach, popular in the machine vision community, bypasses explicit segmentation by identifying a sparse set of “keypoints” – features that are both informative and invariant under different scenes or views of the object (Lowe, 1999, 2004). With a good set of keypoint features, it is possible to match them against a database to do recognition that is robust to changes in scale, rotation, and background. An analogous approach in speech and audio recognition is “glimpsing” (Miller and Licklider, 1950; Cooke, 2006). Here instead of attempting to separate the source from the background by auditory stream segmentation, one attempts to identify spectro-temporal regions where the source target is not affected by the background sounds. This can be effective when both signals are sparse, such as in mixed speech, when only a subset of features is necessary for recognition.

#### Object recognition

Any given scene contains a multitude of objects, so the process of scene analysis is often interrelated with object recognition. In computer vision, object recognition is usually treated as a labeling problem in which each object within a scene is assigned a semantic label and a bounding box that specifies its 2D location in the image. Object recognition is sometimes generalized to “scene understanding” in the field of computer vision, where the task is to segment and label all the different parts of the scene, i.e., all the objects and background, often in a hierarchical manner. The standard approach to solving recognition problems is based on extracting 2D image features and feeding them to a classifier, which outputs the object category. Despite some degree of success over the past decade, these methods have not provided much insight into object recognition and scene analysis as it occurs in animals. One problem is that the task of recognition has been defined too narrowly – primarily as one of mapping pixels to object labels. A label, however, is not sufficient to drive behavior. Many behaviors require knowing an object’s 3D location, pose, how it is situated within the scene, its geometric structure (shape), and other properties needed for interacting with the object.

Another problem with casting recognition as a labeling or categorization problem is that the issue of representation is rarely addressed. Animals, including humans, likely recognize objects using representations that encode the 3D structure of objects in some type of viewpoint invariant form (Biederman, 1987; Edelman, 1999; Kersten et al., 2004). Recent research in the computer vision community has begun to form 3D representations directly using laser scanners (Gould et al., 2008). This improves recognition rates and makes segmentation easier but falls far short of biological relevance, because the representation of 3D structure is still based on point clouds in Euclidean space. Biological representations are likely to be adapted to the structure of natural shapes but currently we do not have models of such representations. Such models could provide testable hypotheses and valuable insights into the nature of the structural representations in biological systems.

### SUMMARY

What emerges from the discussion above is that scene analysis in natural settings encompasses several types of computational problems. While one cannot give a precise definition, it is possible to identify some common principles, which we will explore and develop further below. The first is the importance of solving ill-posed problems, which is very different from the paradigm of (simple) feature extraction underlying many current approaches. Extracting specific information from a complex scene is inherently ambiguous and is only soluble with strong prior information. Another is the importance of grounding representations in 3D space – as opposed to the intrinsic coordinate system of the sensory array – in a manner that drives behavior. Scene analysis must also actively integrate information over time, e.g., by directing the eyes in order to gather specific information for the task at hand, as studied by Land and Tatler (2009). Below we explore these issues further in the context of the scene analysis behaviors of a diverse range of animals.

## SCENE ANALYSIS IN ANIMALS

Against this backdrop of current perceptual and computational approaches to scenes analysis, let us now examine the actual problems faced by animals in the natural environment. We examine four animals in particular which highlight the scene analysis problems solved in different modalities: vision, audition, echolocation, and electrolocation. Because each of these animals must survive in complex environments, they must have developed robust solutions to problems in scene analysis (Bee and Micheyl, 2008). Thus, *studying these and other animals provides a means to learn what is required for scene analysis in the natural environment*. What problems do animals need to solve to carry out their natural behaviors? What are their limits and capabilities, the strategies they use, and the underlying neural circuits and representations?

While the field of neuroethology has long studied a wide range of animal systems, issues relevant to scene analysis have received much less attention. A basic premise of neuroethology is to study a system that is specialized in a behavior of interest, e.g., sound localization in barn owls, animals that forage by listening to the sounds generated by prey. A rich history of research has revealed much about basic perceptual cues and their underlying neural correlates (e.g., timing and intensity differences in sound localization), but, as with many systems, issues in scene analysis have only begun to be addressed. Most studies are designed to manipulate perceptual cues in isolation, which do not require scene analysis, and it has yet to be determined whether such results generalize to more complex natural settings. The history of computational approaches discussed above would suggest that they do not, because the introduction of scene analysis opens a whole new class of problems. Some behaviors can be guided by a few simple cues, e.g., instinctive “key” stimuli eliciting specific responses; others are more complex and require sophisticated analysis. The animals we have chosen provide concrete examples of systems that have to solve difficult problems in scene analysis. We do not yet know the extent to which animals are able to perform scene analysis, because key investigations have yet to be conducted.

For each animal model described below, we consider the range of problems they need to solve, and the extent to which current findings inform us about these animals’ perception of natural scenes and general issues in scene analysis. The point we pursue is a neuroethological one, namely that commonalities in diverse systems can often provide insight into fundamental problems that are of broad relevance.

### VISUAL SCENE ANALYSIS IN THE JUMPING SPIDER

**FIGURE 1:**
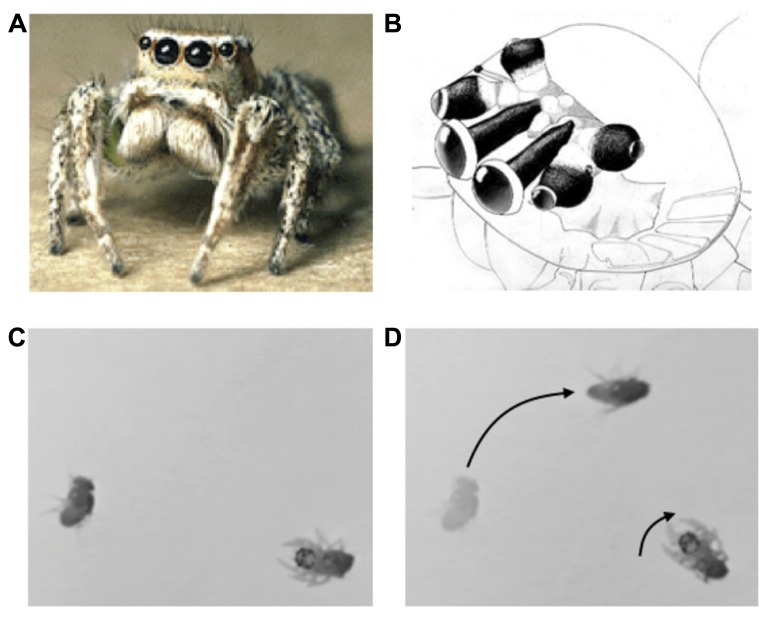
** (A)** Jumping spider (*Habronattus*), **(B)** jumping spider visual system, showing antero-median, antero-lateral, and posterior-lateral eyes (A,B from Tree of Life, Copyright 1994 Wayne Maddison, used with permission). **(C,D)** Orienting behavior of a 1-day-old jumping spider stalking a fruit fly. Adapted from video taken by Bruno Olshausen and Wyeth Bair.

The jumping spider (**Figure 1A**) exhibits a wide variety of visually mediated behaviors that exemplify many of the key problems of scene analysis. In contrast to other spiders, which use a web to extend their sensory space, the jumping spider relies mainly upon its highly elaborate visual system to scan the environment and localize prey, to recognize mates, and to navigate complex 3D terrain. In fact it exhibits many of the same attentional behaviors of predatory mammals (Land, 1972; Jackson and Pollard, 1996).

The visual system consists of four pairs of eyes (**Figure 1B**): one pair of frontal facing principal eyes (antero-median eyes) provide a high-resolution image over a narrow field of view, while the other three pairs provide lower resolution images over a wider field of view and are mounted on different parts of the head so as to provide 360° coverage of the entire visual field (Land, 1985). Interestingly, the retinae of the antero-median eyes are highly elongated in the vertical direction so as to form a 1D image array. These retinae move from side to side within the head in a smooth (approximately 1 Hz) scanning motion that sweeps its 1D retinae across the object of interest (Land, 1969). The jumping spider uses its wide-field, low resolution system to detect moving targets or objects of interest, and then orients its body to focus the high resolution antero-median eyes for more detailed spatial analysis via scanning. In mammals these dual aspects of visual function are built into a single pair of eyes in which resolution falls off with eccentricity, whereas in the jumping spider they are subdivided among different eyes. Note that such multiple eye arrangements are not unique to jumping spiders, but can be found in other animals such as the box jellyfish, which has a total of 24 eyes surveying different parts of the scene (Nilsson et al., 2005; O’Connor et al., 2009). The fact that invertebrates with limited brain capacity “transfer” the scene coverage to eyes with different optical properties is strong evidence of the importance of sensor fusion. Scene analysis is not simply a matter of looking at *the* *image* that falls upon the retina; rather, the brain must assemble disparate bits and pieces of sensory information (in this case, from different eyes) into a coherent representation of the external environment that is sufficient to drive actions.

#### Active perception, segmentation, and tracking during prey capture

Jumping spiders feed mainly upon flies and other insects or spiders. Hunting behavior consists of three steps: (1) a moving object is detected and elicits an orienting response (see **Figures 1C,D**). (2) The target object is then analyzed by the high-resolution, antero-median eyes by scanning. If the object moves during this period the antero-median eyes will also move to track it in a smooth pursuit motion. (3) If the target object is determined to be potential prey, the jumping spider will then engage in a stalking behavior in which it slowly advances forward, crouched to the ground, presumably to avoid detection, prior to raising its front legs and pouncing on the object. Thus, the jumping spider has different behavioral states that dramatically alter its perceptual processing and consequent actions.

These behaviors illustrate an active process of scene analysis, whereby the high resolution eyes – which have narrow field of view – are steered toward a salient item detected by the other eyes. As the object moves, the head and eyes move as appropriate to track the item and keep it in the field of view for high resolution analysis. The scanning motion of the antero-median retinae is used to determine what the object is (see below) and elicit the appropriate action. For a prey item, the spider must estimate the distance for pouncing (possibly using the antero-lateral eyes which have binocular overlap). For all of these tasks, *the prey item must be appropriately separated from the background which is likely to be highly cluttered and contain other moving objects*. The use of multiple eyes to mediate one coherent set of actions also illustrates an integrative process of scene analysis, whereby information from different sources (in this case, different eyes) is combined toward a common goal. How this is accomplished is not known, but the neuroanatomy shows that while each eye has its own ganglion for initial processing, the signals from these different ganglia eventually converge within the jumping spider’s central brain.

#### Object recognition in mate selection

Jumping spiders exhibit a stereotypical courtship behavior in which the male performs a “dance” – consisting of waving the legs or moving from side to side in a specific manner – to attract the attention of a female and gain acceptance prior to copulation. It has been shown that purely visual cues are sufficient to induce these dances. Drees (1952) used a collection of line drawings to find the nominal visual cues necessary to induce courtship behavior and found that the most effective stimulus was in fact a drawing that depicts the most salient features of a spider – a body with legs. It has also been shown that the video image of a conspecific female is sufficient to trigger courtship behavior, and the video image of a conspecific male performing a dance is sufficient to elicit receptivity behavior in the female (Clark and Uetz, 1990).

These studies demonstrate that the male jumping spider performs complex visual pattern recognition in order to detect the presence and assess the suitability of the female. Females must be capable of at least as much sophistication as they must also perceive and recognize the dance movements of the male. Each party must also maintain its attention during this interaction by holding its gaze (antero-median eyes) on the other. Importantly, since the image of each spider subtends a 2D area, the scanning motion of the 1D retinae is crucial to recognition. It has also been observed that the range of scanning is matched to the visual extent of the target (Land, 1969). This scanning strategy again exemplifies an active process of scene analysis, whereby the representations necessary for recognition are built up by moving the sensor array across the scene. It also exemplifies an integrative process, whereby the time-varying photoreceptor activities that result from scanning are accumulated into a stable representation of the object of interest. The image of potential mates must also be separated or disentangled from the background to be seen. Many jumping spiders have colorful markings, presumably for this purpose, but under what conditions of background clutter or occlusion, or under what variations of distance and pose, spiders can be successfully recognized has not been investigated.

#### 3D scene analysis during spatial navigation

Jumping spiders hunt in complex 3D environments in which there may not be a straightforward path for reaching a targeted prey item. When hunting within foliage, for example, the spider may find and localize prey on another branch or object that is not within direct jumping reach, but which requires taking a detour. This detouring behavior has been studied extensively in the species *Portia fimbriata*. It appears that these spiders are capable of analyzing complex 3D layouts in their environment that allow them to plan and execute the proper route to reach prey, even when it requires initially moving away from the target. Tarsitano and Jackson (1994) studied this behavior by placing a prey item in one of two trays that are reachable only by traversing a bent metal rod rising from the ground plane (e.g., as if perched on the leaf of a plant which can only be reached by climbing up the stalk of the plant). The spider is then placed on a pedestal in the center of the arena and begins scanning its environment from this position by repeatedly fixating its principal eyes on objects in its environment. It then commences movement down from its pedestal to the ground plane, and then toward the rod that leads to the prey item irrespective of whether it is in the opposite direction or on the opposite side of the arena. The spider continues its pursuit even though the prey item is no longer visible once the spider moves to the ground. It has also been shown that while en route to a prey item, a jumping spider will occasionally re-orient toward the item by turning its head, and that the angle of these re-orienting turns matches the correct, updated position of the prey item given the distance traveled (Hill, 1979).

These behaviors illustrate another important process of scene analysis, which is the ability to form persistent representations of the 3D layout of a scene appropriate for path planning and navigation. The jumping spider must identify not only the target and its direction, but also the ground plane and traversable objects that lead to the target. Planning and executing a movement to the target requires spatial memory and dynamic updating in order to stay on an appropriate path, even when the goal is not in sight and no longer in the original direction seen.

It is impressive – perhaps even surprising – that such cognitive abilities can be found in a “simple” animal. However when one considers the complexity of the dynamic, natural environment and what is required for robust behavior, these scene analysis capabilities would be essential. Importantly, these abilities lie far beyond what may be achieved by modern computer vision or robotic systems, especially in terms of robustness, which is a testament to the complexity of the computational problems that must be solved.

### AUDITORY SCENE ANALYSIS IN SONGBIRDS

Birdsong serves multiple functions, including mate attraction, mate and species recognition, and territorial defense (Ball and Hulse, 1998; Wiley, 2000; Marler and Slabbekoorn, 2004; Catchpole and Slater, 2008), all of which require the analysis of complex acoustic scenes. Songbirds communicate over long distances (50–200 m) in noisy environments, and although acoustic structure of birdsong is adapted to better stand out from the background (Sorjonen, 1986; Nelson, 1988, 1989; Pohl et al., 2009), the acoustic scene is often cluttered with many types of animal vocalizations (Wiley, 2009) – in some rain forests, there can be as many as 400 species of birds in a square kilometer (Catchpole and Slater, 2008). Similar auditory scene analysis problems are solved by other animals: king penguin chicks, *Aptenodytes patagonicus*, use vocal cues to recognize and locate their parents in dense colonies (Aubin and Jouventin, 1998); female frogs face similar acoustic challenges during mate selection (Feng and Schul, 2006; Gerhardt and Bee, 2006). These and other examples in animal communication have many parallels to the classic cocktail party problem, i.e., recognizing speech in complex acoustic environments (Cherry, 1953; Brémond, 1978; Hulse et al., 1997; Bee and Klump, 2004; Bee and Micheyl, 2008).

#### Scene analysis in territorial defense

**FIGURE 2:**
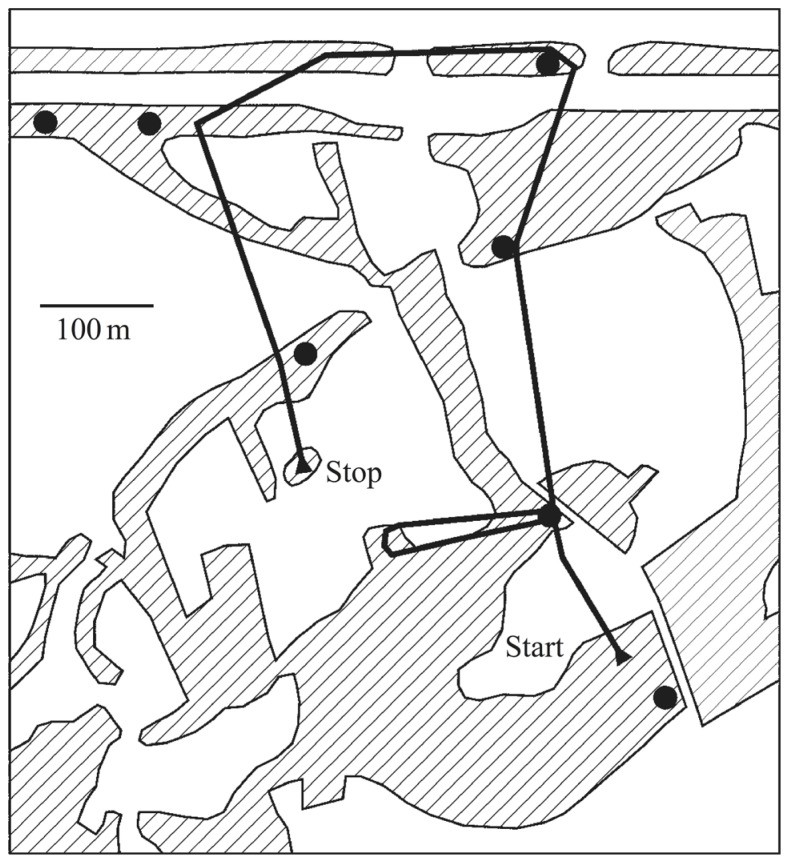
**Territorial prospecting.** Songbirds use song as acoustic territorial markers to serve as a warning to potential invaders and rely on sound for locating other birds in complex acoustic scenes and natural environments. The black dots indicate the positions of established singing territorial males, most of which would be audible from any one position, in addition to numerous other sounds. The black line shows the prospecting path of a translocated and radio-tagged male nightingale. Hatched areas indicate reed, bushes, or woods separated by fields and meadows. Figure from Naguib et al. (2011) which is based on data from Amrhein et al. (2004).

Auditory scene analysis is crucial for songbirds in one of their primary behaviors: territorial defense. Songs are used as acoustic markers that serve as warning signals to neighboring rivals. From acoustic cues alone, songbirds must keep track of both the identities and positions of their territorial neighbors to recognize when a rival has trespassed (**Figure 2**). Localization accuracy in both direction and distance is important because if they do not fight off an invader they risk losing ground, but an excess of false alarms or poor estimates would waste time and energy.

The accuracy and robustness of species-specific song recognition and localization can benefit from higher-level knowledge of song structure. Songbirds can localize acoustic sources in natural habitats accurately even in noisy environments (Klump, 2000). The precise acoustic cues songbirds use remain unclear. Their small head size provides minimal timing and intensity differences normally used for lateralization judgments, and the reliability of these cues does not predict their level of accuracy in natural settings (Nelson and Stoddard, 1998). One explanation is that the birds can make use of higher-level knowledge to help disambiguate the acoustic cues (Nelson and Stoddard, 1998; Nelson, 2002; Nelson and Suthers, 2004). It is also likely that songbirds make use of an interaural canal to localize sound sources (Klump and Larsen, 1992; Larsen, 2004). Songbirds (and other birds) also exhibit the precedence effect which may serve to minimize interferences from echoes and reverberation (Dent and Dooling, 2004; Spitzer et al., 2004; Spitzer and Takahashi, 2006). Sound localization in songbirds may employ all of these mechanisms and requires some degree of scene analysis, because the acoustic cues for localization must be separated from the clutter of other sounds and the background noise.

Estimating the distance of singing birds is difficult because it is not clear if this information can be derived from generic acoustic cues (Morton, 1986; Naguib and Wiley, 2001; Brumm and Naguib, 2009). To judge distance of a conspecific, a bird must assess the level of degradation in a song (in terms of frequency-dependent attenuation and reverberation) after it has propagated through the environment. This suggests that songbirds make use of higher-level knowledge, and there is evidence that songbirds are more accurate in ranging familiar songs (Morton, 1986, 1998a,b; Shy and Morton, 1986; Wiley, 1998; Morton et al., 2006).

A representation of the territorial space is necessary for locating and tracking the positions of other songbirds, and it must also properly account for the bird’s own orientation and movement within the territory. Songbirds can remember the spatial location of an intruder and can accurately estimate its range even after playback has ended (Morton et al., 2006). This representation is also likely to be integrative because conditions are noisy, and it is not always possible to localize accurately from a single instance of song. Any form of triangulation from multiple instances of a rival song from different locations would also require an integrative representation. There is evidence that songbirds experience spatial unmasking when overlapping sounds arrive from different directions (Dent et al., 2009), suggesting that they can perform scene analysis using both acoustic features and spatial location of sound sources.

#### Song recognition and auditory source separation

Songbirds recognize the songs of their territorial neighbors, responding more aggressively to unfamiliar strangers (Bee, 2006), and they retain this ability in the presence of acoustic clutter such as the songs of other birds (Hulse et al., 1997; Wisniewski and Hulse, 1997; Appeltants et al., 2005). This also requires a representation of song structure which is likely to be learned because European starlings show persistent memory for both tonal signals and amplitude modulations (Zokoll et al., 2007, 2007). Starlings also show long-term memory for individuals (Braaten, 2000) suggesting their representation of acoustic targets (i.e., other songs) is highly adaptive.

A number of studies have investigated the extent to which songbirds can process song in the presence of background noise and interfering acoustic clutter. Brémond (1978) used speakers to broadcast conspecific songs of wrens within their territorial boundaries. The normal territorial response was largely unaffected when the songs were masked with a variety of equally loud stimuli, including heterospecific songs, a stimulus composed of randomized 50 ms fragments of conspecific song, or a mixture of eight natural wren songs. None of the maskers presented in isolation elicited a response, suggesting that the wrens were adept at identifying an intruding song even in the presence of a significant amount of acoustic clutter. These abilities do have limits, however. An earlier study of species recognition by Brémond (1976) found that Bonelli’s warblers could not identify their own song when it was masked with inverted elements from the same song.

**FIGURE 3:**
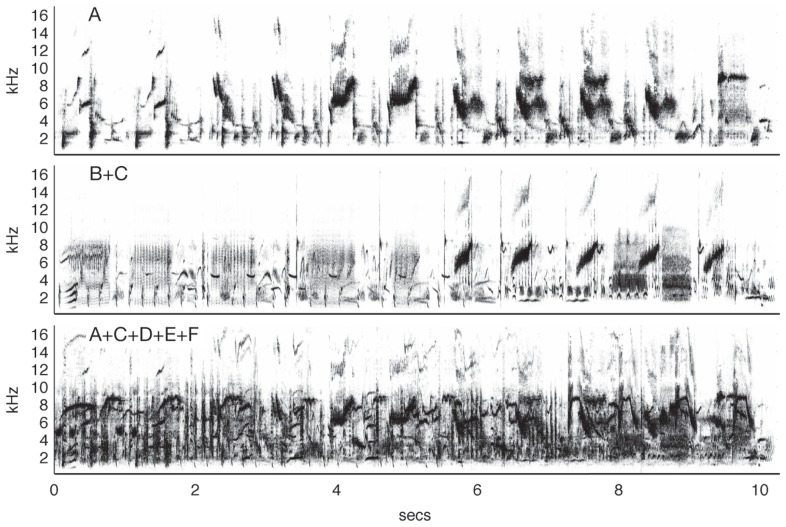
**Auditory source separation by starlings.** The top panel shows a spectrogram from a 10-s segment of typical starling song (*Sturnus vulgaris*). In an experiment by Wisniewski and Hulse (1997), starlings were trained to discriminate one of 10 song segments produced by Starling A from 10 song segments produced by Starling B. The birds maintained discrimination when the target song was mixed with a novel distractor song from Starling C (middle) and in the presence of four novel conspecific songs (bottom), a feat which human listeners could not do, even after training. Figure after Wisniewski and Hulse (1997) using songs from the Macaulay Library of the Cornell Lab of Ornithology.

A series of experiments by Hulse and colleagues (Hulse et al., 1997; Hulse, 2002) showed that European starlings could accurately recognize conspecific song (demonstrated by key pecking) in the presence of a variety of other songs and a noisy dawn chorus. Importantly, the birds were trained so they never heard the target song type in isolation, i.e., the starling songs were always mixed with a song of another species. The birds then had to compare this pair to another mixed song pair of two different species. Not only could the birds accurately classify the song pairs that contained starling song, but they also generalized with no additional training to song pairs with novel songs and to songs presented in isolation. Further studies (Wisniewski and Hulse, 1997) showed that the starlings were also capable of accurately discriminating song segments from two individual starlings, even when each was masked with song segments from up to four other starlings (**Figure 3**). These studies suggest that the birds were not perceiving the song pairs as fused auditory objects and learning the feature mixtures, but recognized the target song by segregating it from other acoustic stimuli with very similar structure. Similar results have been observed in zebra finches (Benney and Braaten, 2000).

#### Active perceptual behaviors

Songbirds also take a variety of actions to facilitate scene analysis (Brumm and Naguib, 2009). They perch in locations and positions that maximize the range of the acoustic signal (while still avoiding predation); they sing more repetitions when there is higher background noise; they also avoid overlapping their songs (Brumm, 2006). These compensatory behaviors are not unique to songbirds, but are also shared by other animals that rely on acoustic communication such as penguins and frogs (Aubin and Jouventin, 1998; Murphy, 2008).

The broad range of songbird behavior carried out in complex acoustic environments strongly suggests that songbirds successfully perform several aspects of scene analysis. There also remains the possibility that songbirds achieve these feats via simpler means. For example, songbirds might recognize song by key features or “glimpsing” (Miller and Licklider, 1950; Cooke, 2006), where it is only necessary to get an occasional unmasked “glimpse” of some part of the song in order to recognize it. This could be tested by controlling the masking songs in a systematic way, but doing so requires a detailed model of the song recognition process which has remained elusive. It seems more likely that for recognition (and mate selection by females), the acoustic elements of song need to be not only separated from the clutter of other sounds and the noise of the background but also grouped correctly over time. Supporting this is the observation that songbirds exhibit temporal induction of missing song segments (Braaten and Leary, 1999). The intricate structure of birdsong and the growing evidence that songbirds use higher-level structural knowledge suggest that songbirds perform auditory scene analysis and spatial auditory perception in a manner that is analogous to contextual inference and auditory grouping in speech recognition. Furthermore, spatial memory, path planning, and active perception are essential aspects of territorial defense and mate selection. Together these span many general aspects of scene analysis discussed above and highlight new ways songbirds could be studied to gain general insights into scene analysis.

### ACTIVE AUDITORY SCENE ANALYSIS IN BATS

Studies of echolocating bats can shed light on general problems of scene analysis that lie far outside the realm of human experience. These animals employ a high resolution, active sensing system to extract information from the natural environment based on the echoes from calls emitted by the bat. The features of a bat’s echolocation calls impact the acoustic information it receives to build its auditory scene, and therefore, detailed study of sonar call parameters provides direct access to the signals used by an animal to perceive its 3D environment. Importantly, the bat’s active motor adjustments reveal how the animal deals with ill-posed perceptual problems, where echo snapshots carry incomplete and ambiguous information about the natural scene.

The high spatial resolution and active components of bat echolocation offer a special opportunity to (1) identify general principles of scene analysis that bridge hearing and vision, and (2) analyze the very acoustic signals used by bats to represent the natural scene, which can, in turn, inform principles of scene analysis in auditory generalists, including humans. We elaborate below on these key features of echolocation as we summarize empirical findings from the literature.

The bat’s sonar scene consists of echoes reflecting from targets (flying insects, stationary fruit, or other food items) and clutter (vegetation and other objects) and background (the ground). Oftentimes the echoes the bat encounters from a complex scene contain incomplete or ambiguous information about object features and location: (1) cascades of overlapping echoes from targets and clutter may be difficult to assign to corresponding sonar objects, (2) very short duration echolocation calls return information about a dynamic environment within only a restricted slice in time, (3) directional sonar calls return information from a restricted region in space, (4) rapid attenuation of ultrasound limits the operating range of echolocation, and (5) signals produced by nearby bats and other animals can interfere with the processing of echoes, but perhaps also be exploited for extra information. Nonetheless, echolocating bats overcome these challenges to successfully navigate and forage using biological sonar.

#### Spatial scene perception

**FIGURE 4:**
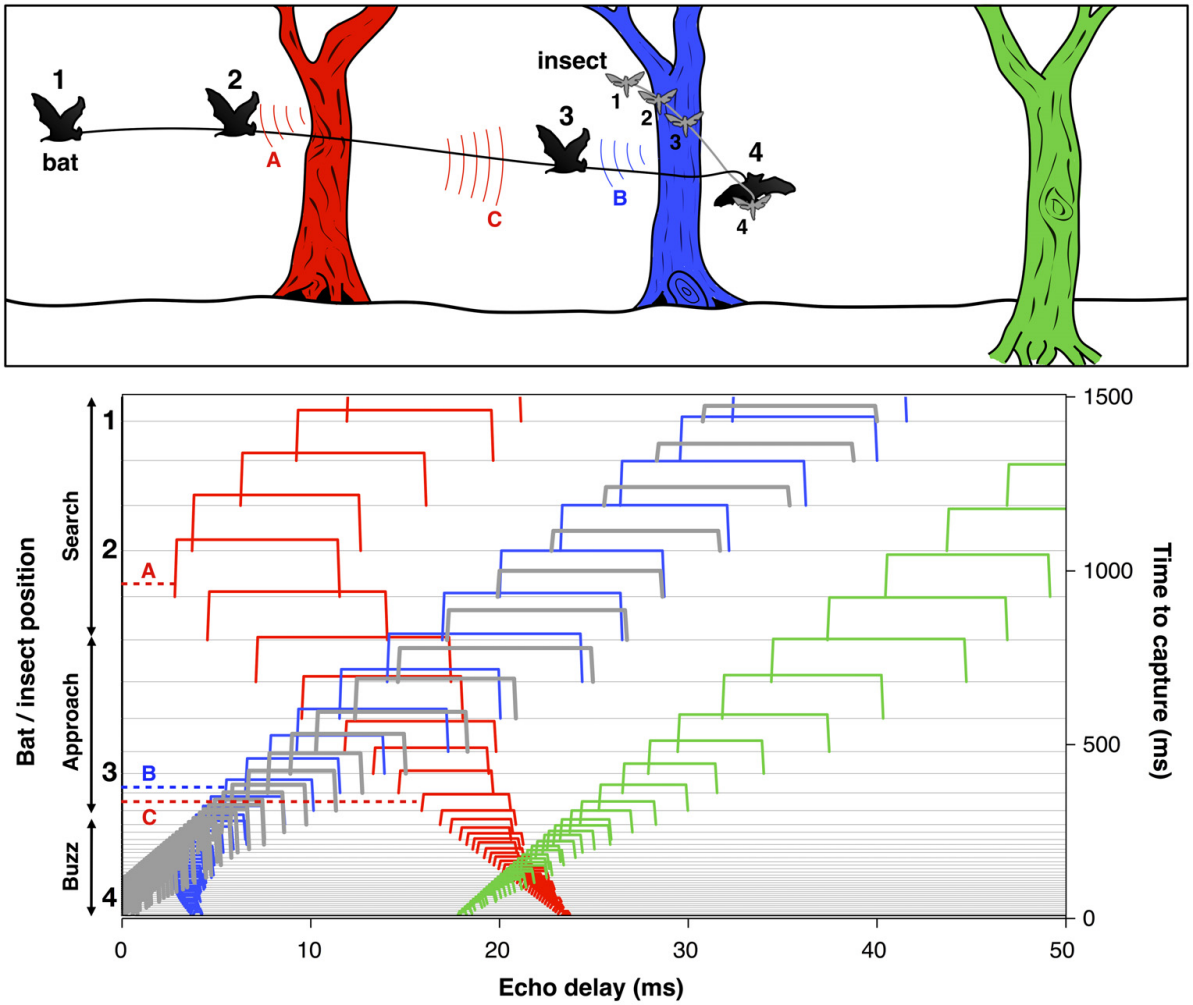
**Bat scene analysis.** Schematic illustrating how echoes from different objects in the path of the bat’s sonar beam form acoustic streams with changing delays over time. *Upper panel*: The cartoon shows a bat pursuing an insect in the vicinity of three trees at different distances. The numbers indicate the positions of the bat and insect at corresponding points in time. Color-coded arcs illustrate an echo from the red tree at position 1 (A) and echoes from the blue and red trees at position 3 (B and C). *Lower panel*: Echoes from the insect (thick gray lines) and each of the trees (red, blue, and green) arrive at changing delays (*x*-axis) over time (right *y*-axis) as the bat flies in pursuit of its prey. Each sonar vocalization (not shown) results in a cascade of echoes from objects in the path of the sound beam, which arrive at different delays relative to vocalization onset. Time to capture proceeds from top to bottom. At time 0, the bat captures the insect. The numbers 1–4 to the left of the *y*-axis indicate the times of the corresponding bat and insect positions in the cartoon. The thin horizontal gray lines display the echo returns from successive vocalizations which change in duration as the bat moves from search to approach to terminal buzz phases (left *y*-axis). Echoes are displayed as color-coded open rectangles to illustrate the relative arrival times from the insect and each of the trees. The letters A, B, and C link selected echoes to the arcs in the cartoon above. The duration of echoes, indicated by the width of rectangles, changes proportionately with the duration of sonar calls and appear as narrow ridges when call duration is very short during the approach and buzz phases. Note that the delay of echoes from the red tree and blue tree initially decrease over time, and later increase after the bat flies past them. Adapted from Moss and Surlykke (2010).

To successfully track a selected prey item and avoid collision with other objects, the bat must localize and organize complex 3D acoustic information and coordinate this representation with motor planning on very rapid time scales. For example, when a bat is seeking insect prey in the vicinity of vegetation, each sonar call returns echoes from the target of interest, along with echoes from branches, leaves, and other objects in the vicinity (**Figure 4**). The resulting echo streams carry information about the changing distance and direction of objects in space. By integrating information over time, the bat can sort and track target echoes in the midst of clutter. This scene analysis task is aided by their active control over sonar call design, along with head and pinna position. Some bats, for example, *Myotis septentrionalis* (Miller and Treat, 1993) or *Myotis nattereri* (Siemers and Schnitzler, 2004) use very short and often extremely broad band frequency modulated (FM) calls to sharpen up the representation of closely spaced objects, which enables them to distinguish prey from clutter. The nasal-emitting bat *Micronycteris microtis* (Phyllostomidae) can detect and seize completely motionless prey sitting on leaves; this is among the most difficult auditory segregation tasks in echolocation, which may rely on learning and top-down processing (Geipel et al., 2013). In addition, recent psychophysical studies suggest that bats using FM calls may experience acoustic blur from off-axis echoes, due to frequency-dependent directionality of sonar signals and the dependence of auditory response latencies on echo amplitude. This off-axis “blur” could serve to minimize clutter interference in the natural environment (Bates et al., 2011).

Bats that produce long constant frequency signals combined with short FM sweeps (CF–FM), such as the greater horseshoe bat, *Rhinolophus ferrumequinum*, solve the problem of finding prey in dense vegetation by listening for Doppler frequency and amplitude modulations in echo returns that are introduced by the fluttering wings of insect (Schnitzler and Flieger, 1983; von der Emde and Schnitzler, 1986; von der Emde and Menne, 1989). They can also use this acoustic information to recognize insect prey (von der Emde and Schnitzler, 1990), suggesting that auditory scene analysis by echolocation builds on prior knowledge.

#### Active perception in sonar scene analysis

The fast, maneuverable flight of bats requires not only a detailed representation of the natural scene to discriminate between foreground (prey, conspecifics, and clutter) and background (landscape, ground, large structures like trees, houses, rocks), but also very rapid updates to take into account their own movements, as well as those of the prey and conspecifics. How does the bat accomplish this daunting auditory scene analysis task with ambiguous echo information and on a millisecond time scale? Part of the answer to this question lies in this animal’s adaptive vocal-motor control of sonar gaze and frequency.

#### Scene analysis through gaze control

Detailed analyses of the big brown bat’s echolocation behavior has revealed that this animal sequentially scans auditory objects in different directions, by moving the axis of its sonar beam and inspects objects at different distances, by making range-dependent adjustments in the duration of its calls (Ghose and Moss, 2003; Surlykke et al., 2009; Falk et al., 2011). Bats also adjust the width of the sonar beam to the situation, to use a broader “acoustic field of view” close to clutter than out in the open where a narrow “long range” beam is advantageous (Jakobsen et al., 2013). The bat’s active adjustments in the direction and distance of its sonar “gaze” help the bat resolve perceptual ambiguities in the sonar scene by sampling different regions in space. Sonar beam aim also indicates where in space the bat is attending, and suggests parallels with eye movements and visual gaze (Land and Hayhoe, 2001). This observation also suggests that the bat uses working and short-term memory to assemble a spatial representation of the environment from a series of echo snapshots from different locations (Moss and Surlykke, 2001; Surlykke et al., 2009). It has also been demonstrated that pinna movements of echolocating bats that use CF sonar signals serve to enhance echo information for spatial localization (Mogdans et al., 1988; Gao et al., 2011). Together, the bat’s active control over sonar call features, head direction, and pinna position contribute to solving the computational problem of sorting sounds arriving from different directions and distances.

#### Scene analysis through sound frequency control

When bats forage together in groups, they face a “cocktail party” challenge of sorting echoes generated by their own sonar calls from the signals and echoes of neighboring bats. A recent laboratory study investigated this problem by studying the acoustic behavior of pairs of echolocating big brown bats (*Eptesicus fuscus*) competing for a single prey item (Chiu et al., 2009). The results of this study show that the bat makes adjustments in the spectral characteristics of its FM calls when flying with conspecifics. Importantly, the magnitude of these adjustments depends on the baseline similarity of calls produced by the individual bats when flying alone: bats that produce sonar calls with similar spectrum (or frequency structure) make larger adjustments in their sonar calls than those bats whose baseline call designs were already dissimilar. This suggests that simple frequency cues may be sufficient to reduce perceptual ambiguities, and the separation of frequency features of sonar calls produced by different bats aids each individual to segregate echoes of its own sonar vocalizations from the acoustic signals of neighboring bats (see Ulanovsky et al., 2004; Bates et al., 2008).

Hiryu et al. (2010) report that big brown bats flying through an array of echo reflecting obstacles make frequency adjustments between alternating sonar calls to tag time dispersed echoes from a given sonar call with spectral information. Many other bats normally alternate between frequencies from call to call (Jung et al., 2007). These findings suggest that bats may treat a cascade of echoes following each sonar vocalization as one complete view of the auditory scene. If the integrity of one view of the acoustic scene is compromised by overlap of one echo cascade with the next, the bat changes its call frequencies to create the conditions for segregating echoes associated with a given sonar vocalization, thus providing additional evidence for the bat’s active adjustments in signal frequency to resolve ambiguity in assigning echoes from objects at different distances.

#### Broader perspectives on scene analysis offered by echolocation

Echo reflecting objects are in effect sound sources, whose acoustic characteristics are shaped by the bat’s active control over its sonar signals. The bat’s active sensing allows us to directly measure the signals used by an animal to resolve perceptual ambiguities that arise in scene analysis problems. Furthermore, the bat’s adaptive adjustments in sonar call direction, intensity, duration, timing, and frequency emphasize the importance of these acoustic parameters to specific scene analysis tasks, and suggest parallel processes for the cues used in natural scene perception by other animals, including humans.

### SCENE ANALYSIS IN THE ELECTRIC FISH

**FIGURE 5:**
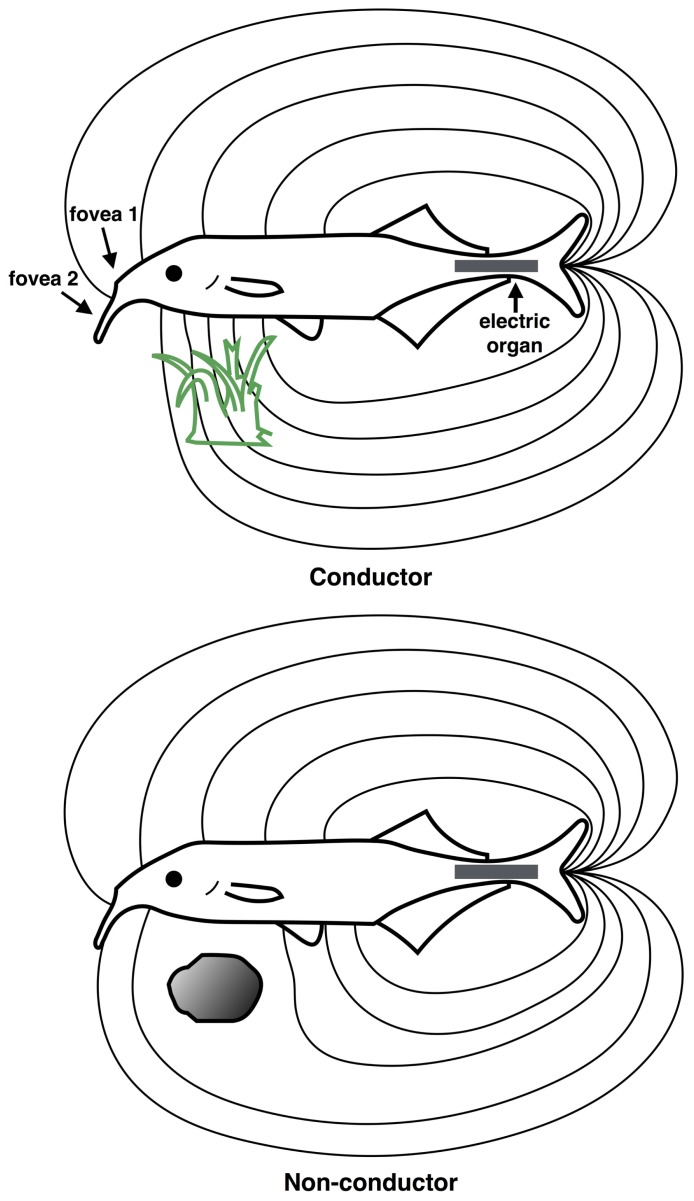
**Scene analysis in electroreception.** The “electric image” of the external environment is determined by the conductive properties of surrounding objects. The electric field emanates from the electric organ in the tail region (gray rectangle) and is sensed by the electroreceptive skin areas, using two electric “foveas” to actively search and inspect objects. Shown are the field distortions created by two different types of objects: a plant that conducts better than water, above (green) and a non-conducting stone, below (gray). (Redrawn from Heiligenberg, 1977).

As with the echo-locating bat, the manner in which electric fish perceive the surrounding environment provides a clear example of scene analysis principles at work that is divorced from human introspection. The sensory world of the electric fish consists largely of distortions of its self-generated electric field, in addition to the electric fields generated by other fish (Nelson, 2011). Although still equipped with a visual system, electroreception has been shown to be the dominant sense used for foraging, orientation and communication tasks for these animals. The electrical environment contains targets such as prey items or other fish which must be detected against complex backgrounds, and it must navigate through complex terrain (see **Figure 5**).

In mormyrids, the electric field is generated by the electric organ residing in the caudal peduncle (tail region), which generates a relatively uniform electric field over the anterior body surface where most electroreceptors are located (von der Emde, 2006). An “electric image” of the external environment is formed on the electroreceptor array according to how physical objects distort the electric field, as shown in **Figure 5**. An object that is a good conductor relative to water will cause electric field lines to bunch up, creating a positive difference in the electric field on the corresponding portion of the electroreceptor array. Conversely, a poor conductor relative to water will cause electric fields lines to disperse, creating a negative difference in the electric field pattern on the electroreceptor array.

In addition to conductance, the capacitive properties of an object may also be ascertained by how it changes the waveform of the electric organ discharge (EOD). The EOD itself is composed of a series of pulses, each of which has a characteristic waveform, typically less than 1 ms duration. In mormyrids, a copy of the EOD signal is sent to electrosensory areas of the brain. Thus, it is possible for the animal to directly compare the sensed signal with that which was actually generated. An object with low or no capacitance, such as a non-living object, will leave the waveform shape unaffected. Most living objects however, such as insect larvae, other fish, and plants possess complex impedances, and so they will significantly alter the waveform shape, which behavioral studies show is detectable by the animal (von der Emde, 2006).

Due to the high conductivity of water, the range over which the electric fish can sense objects is only a few centimeters. Nevertheless, electroreception mediates a wide range of scene analysis behaviors important to the animal’s survival, which we describe here.

#### Object recognition in electric scenes

The mormyrid’s object recognition and discrimination abilities have been explored through behavioral studies (von der Emde and Schwarz, 2002; von der Emde, 2004; von der Emde et al., 2010). By assessing performance on simple association tasks, it has been shown that electric fish are capable of discriminating the shape of objects (e.g., cube vs. pyramid), even against complex and variable backgrounds. Doing so is non-trivial because the electric fields from multiple objects will superimpose and create a seemingly complex electric image on the electroreceptor array. Thus, the animal must solve a figure-ground problem similar to that in vision or audition, in which the sensory contributions of background or clutter must be discounted in order to properly discern an object. Perhaps even more impressive is the fact that the animal can generalize to recognize different shapes independent of their material properties (metal or plastic) or distance. It can discriminate small from large objects, irrespective of distance. Thus, the animal is capable of extracting invariances in the environment from the complex electroreceptor activities – i.e., despite variations due to material properties or distance, it can nevertheless make correct judgments about the shape and size of objects.

#### Active perception during foraging

When foraging for food, mormyrids utilize their two electric “foveas” in an active manner to search and inspect objects. The two foveas are composed of a high density region of electroreceptors, one on the nasal region, and the other on the so-called *Schnauzenorgan* (Bacelo et al., 2008). Unknown objects are first approached and inspected by the ventral nasal organ, and then more finely inspected by the Schnauzenorgan (von der Emde, 2006). When foraging, the animal engages in a stereotypical behavior in which it bends its head down at 28° such that the nasal fovea is pointing forward or slightly upward, and it scans the Schnauzenorgan from side to side across the surface to search for prey. When a prey item is detected (presumably from its capacitive properties) it is inspected by the Schnauzenorgan before the fish sucks in its prey. Thus, the animal must correctly interpret the highly dynamic patterns of activity on the sensory surface in accordance with this scanning movement in order to properly detect and localize prey. This is an example of an active process demanding the coordination of perception and action.

#### Spatial navigation

Mormyrids are frequently seen swimming backward, and they avoid obstacles with ease, finding their way through crevices in rocks (Lissmann, 1958). Presumably these abilities are mediated by the electric sense, since the eyes, which are poorly developed, are at the front of the animal. They are also known to navigate at night in complete darkness (von der Emde, 2004). Thus, it would appear that electric fish can obtain a sufficient representation of 3D scene layout from the electric field in order to plan and execute maneuvers around objects. How accurate and what form this representation takes is not known, but it has been shown through behavioral studies that they can judge the distance to an object from the spatial pattern across the electroreceptor array (von der Emde, 2004). A hypothesized mechanism for doing this is by calculating the slope to amplitude ratio, i.e., the rate of change in the electric field across the surface divided by the maximum.

#### Communication in electric scenes

In addition to sensing distortions in the electric field caused by other objects, electric fish also detect the electric fields generated by other fish. In mormyrids, the waveform of the EOD is used for communicating species, sex, and social status, while the sequences of pulse intervals (SPIs) is used for communicating rapidly changing behavioral states and motivation (Carlson, 2002; Carlson and Hopkins, 2004; Wong and Hopkins, 2007). During the breeding season, males of many species have a longer EOD than females and often have a sex-specific waveform. During courtship, they may produce high-frequency bursts termed “rasps,” while during overt aggression they may produce “pulse pairs.” Conditioning experiments demonstrate that they are also able to distinguish individual differences in the EOD, supporting a potential role in individual recognition. Thus, a rich array of structural information regarding the identity and intentions of other animals is available in the EOD, and this structure must be properly extracted and analyzed in order to make appropriate behavioral decisions. Importantly, these signals must be properly separated from the background variations in the electric “scene” used for detecting prey and navigation, in addition to the contributions of multiple surrounding fish.

## COMMON PRINCIPLES IN NATURAL SCENE ANALYSIS

The animals discussed above exhibit a wide variety of scene analysis capabilities that enable robust behavior in complex environments. What lessons can we draw from these examples to guide our study of scene analysis? One, which we shall expand upon below, is that the ability to extract information from complex, natural scenes is paramount, yet far beyond what is commonly addressed in laboratory studies that simplify the stimulus or task. Another is that all of these abilities still lie far beyond current computational algorithms, which means that we lack essential conceptual frameworks for studying them. Just as the principles of optics guides our study of eyes, principles of information processing – *most of which have yet to be discovered* – will be needed to study how scene analysis is carried out in animals.

To distill what we have learned both from the review of current approaches and the discussion of animal capabilities above, we develop a framework around a set of common properties that enable scene analysis in the natural environment:

1. The ability to solve *ill-posed problems* inherent in extracting scene properties from raw sensory inputs
2. The ability to optimally *integrate* and *store* information across time and modality
3. Efficient recovery and representation of *3D scene structure*
4. Optimal *motor actions* that guide the acquisition of information to progress toward behavioral goals

Our understanding of how each of these is accomplished remains incomplete, but we conjecture that each is an essential aspect of the larger problem of scene analysis. These points are further elaborated below.

### COMPONENTS OF A NATURAL SCENE

**FIGURE 6:**
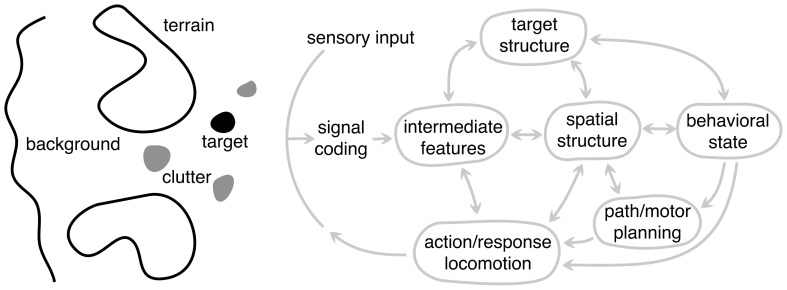
**A schematic framework for scene analysis.** The arc labeled sensory input represents all the sensory information available to the system, possibly from different modalities. To the left of this arc is an abstract representation of the different components of the external scene: target, clutter, terrain, and background, each of which are processed in different ways depending on the particular scene analysis task. These are depicted as being spatially distinct, but they need not be. To the right of the sensory input arc is the general set of processing components (or stages) underlying biological scene analysis. Each node represents a different level of abstraction and is hypothesized to play a distinct role in the overall system but need not correspond to distinct brain areas. Not all animals use every component because animals have a range of perceptual capabilities and specializations. Arrows represent the flow of information between components, with a double arrow indicating that information can flow in both directions. The arrow going to the sensory input arc represents the “action” of the system and the output of the largest dynamic loop. The animal’s motor actions make it progress toward the behavioral goals, but also change the sensory input in order to gain more information about the scene.

Before delving into the properties of scene analysis, it is useful to first spend some time considering the different components of the scene itself (**Figure 6**). We will define these generically so that they apply across modality and to a wide range of animals and tasks. The *target* (black blob) represents an object (or information) in the scene to which the animal’s attention or behavior is directed. This could be a fly for the jumping spider or the song of a rival songbird. It is important to distinguish between the target in the natural scene and what is often dubbed the “sensory stimulus” in the laboratory setting. In a natural setting, the stimulus is presented in the context of the entire sensory scene. The target can also be defined more generally to represent information the animal needs to acquire, e.g., location of the neighboring bird or its fitness. Thus, the target is rarely extracted directly from the sensory input – the necessary information must be *inferred*. *Clutter* (gray blobs) generically represent false targets or other components of the scene that could be confused with the target, such as other flying insects while the bat is pursuing a moth or songs of other birds. This interference vastly increases the complexity of processing, because many problems become ill-posed. This is why camouflage is an effective adaptation. In the extreme case of high clutter and sparse stationary targets, animals face a complex search task, – a natural scene version of a “Where’s Waldo?” game.

For most animals, the ability to approach or pursue a target is as important as locating it. For example, the jumping spider must determine how to traverse the local terrain and navigate around obstacles to get within jumping distance of prey. We refer to these scene components generically as *terrain* (large blobs). The processing and inference of terrain information is very different from that of the target, which is often confined to a small region of the scene. Terrain, in contrast, tends to be more extended. Successful locomotion depends on extracting sufficient information about the terrain shape for accurate foot placement, or sufficient information about the size and location of obstacles for accurate path and movement planning. In vision, terrain is typically the ground, which is usually stationary, but obstacles could be either fixed or moving, such as swaying branches and foliage during echolocation.

The *background* of a scene refers to the remaining structure that is not processed for terrain or for locating the target. While this aspect of the scene might provide useful sensory information for other behavioral functions, from the viewpoint of processing in scene analysis we generally consider the background to be a source of noise that generically degrades the information about the target or terrain, although it can also provide important contextual information that influences perception (Oliva and Torralba, 2007).

### COMPONENTS OF NATURAL SCENE ANALYSIS

We decompose the scene analysis process into a set of general components shown to the right of the sensory arc in **Figure 6**. The diagram takes form of the standard perception–action cycle (von Uexküll, 1926; Gibson, 1979) with some elaborations for scene analysis. Nodes represents different components of analysis, but these do not correspond to distinct neural substrates.

The first level of processing converts the physical energy arising from the scene signals to a neural code. At this level, it is not clear to what extent the computations are specific to scene analysis, but there are animals with sensory organs that are adapted to facilitate scene analysis at the periphery, e.g., the estimation of depth from defocus in the jumping spider using a multi-layer retina (Nagata et al., 2012) or the specialization of cell types in the retina (Field and Chichilnisky, 2007; Gollisch and Meister, 2010; Masland, 2012). We use a single arrow out of the signal coding level to indicate that the information flow is largely in one direction, although it is conceivable that in some cases, feedback to the periphery could play a role in scene analysis by efferent control of early sensory processing or transduction (e.g., olivocochlear feedback in the auditory system). Importantly, coding is just an initial step in acquiring data about the environment – it does not make explicit the properties of interest in the scene. For that, further processing is needed.

#### Inference and prior knowledge

The recovery of information about the scene is an ill-posed inference problem that requires some degree of prior knowledge of scene structure. The level of *intermediate features* is the first stage where scene components and parameters are disentangled. Properties such as the contour of targets that blend in with their backgrounds, the slant and shape of terrain, or the parts of objects missing due to occlusion are not made explicit at the level signal coding, they must be *inferred*.

Although low-level features such as oriented edge detectors can signal boundaries of surfaces having different luminance values, they do not reliably signal boundaries of complex surface textures, such as the boundary between a tree trunk and background elements in a scene (Karklin and Lewicki, 2009). Similarly, retinal disparity is an unreliable cue for depth because computing the binocular correspondence for complex surfaces is often confounded by false matches or missing correspondences, and this is further compounded by multiple objects, complex surface shapes, and scenes with multiple occlusions. Analogous challenges are faced for determining spatial location of sound sources. Inter-aural time and intensity differences or the time of arrival of echoes can provide information about the direction or distance of an isolated sound source, but these cues are also compromised in more complex acoustic environments, which may have multiple sources, reverberation, and significant levels of background noise.

As discussed above, solving these types of ill-posed inference problems ultimately depends on higher-level knowledge, but intermediate-level features provide a staging ground at which perceptual units can be organized, e.g., features originating from different sound sources or the formation of distinct representations of visual surfaces (Barrow and Tenenbaum, 1978). In vision, this stage partly corresponds to what is loosely referred to as perceptual organization, segmentation, or grouping. These terms, however, are more often used to describe the perceptual processing of simple 2D or “idealized” stimuli and rarely get at the problem of how elements of the 3D scene, such as objects and surfaces, are extracted from sensory inputs. The complexity of processing at this stage is closer to the kinds of problems that have been investigated in computer vision, such as shape-from-shading or structure-from-motion, which estimate the 3D structure of a scene or objects. To date, however, these problems have no general solutions for natural scenes.

For simpler aspects of scene analysis, it might be possible to go directly from an intermediate level representation to an action or response (as depicted by the arrow). For more complex tasks, however, feedback in the form of higher-level knowledge is required because the separation of components and inference of scene parameters that occurs at this stage is a highly ill-posed problem in which a multitude of interpretations could be consistent with the sensory features. For example, in the computation of binocular disparity, high-level knowledge of typical 3D structures in the scene makes it possible to arrive at an unambiguous interpretation of local depth cues (**Figure 6**, double arrows).

Higher-level representations can come in many forms, but for our purposes here we single out two general types: *object memory* and *spatial memory*. Note that we use the term “memory” here in a broader sense to mean implicit knowledge or a representation of object structure, which could be innate or acquired through experience. It also encompasses the computational inference and feedback discussed in the previous section. Object memory includes information such as object shape, e.g., the shape of a conspecific jumping spider or acoustic characteristics like song structure or the echo signature of a moth. Spatial memory combines input from multiple modalities (or different sources within a modality) to form a more accurate and robust representation of the scene layout, potential target locations, terrain, and obstacles, for example, the daily path taken by a bat to forage (Tsoar et al., 2011) or spatial structure at more proximal scales (Tommasi et al., 2012). The arrow between object and spatial memories indicates that these processes are not necessarily independent and may be mutually informative, e.g., certain targets occur only in certain locations. Note that the level of detail in these representations in these areas need only be sufficient to meet the behavioral requirements of the system. Furthermore, they need not have an isomorphic relationship to object category or 3D spatial structure but could encode information extracted from the scene and represented in a very reduced dimensionality.

#### Integrative representations of scene structure

An essential aspect of the higher-level target and spatial representations is that they are persistent and integrate information over time and across multiple actions. Note that this is not a literal “visual integrative buffer” or low-level visual memory (see Wade and Tatler, 2005 for a review and critique of this idea). The integration of information is in the Bayesian sense of combining multiple sources of information to infer underlying structure. It is performed at a higher-level and pertains to the basic problem of the inference of scene properties. Without integration and consolidation, the system could only react to whatever sensory information is available at a given instant in time, which is often too ambiguous to drive action. By integrating sensory information over time, the system can build up a representation of the external environment that allows it to more reliably identify objects, more quickly locate targets, or more accurately estimate other aspects of the scene. The integration acts at multiple time scales that vary from relatively shorter – e.g., building up continuously from the movements of the sensors and dynamics of motor actions – to relatively longer, e.g., by building up a synthesized scene representation from sensory information acquired at different locations (Land and Furneaux, 1997; Land, 1999; Tatler et al., 2005; Byrne et al., 2007; Epstein, 2008; Wolbers et al., 2008, 2011).

An example of such integration occurs when locating objects in cluttered environments, which is a basic problem in scene analysis that animals face when pursuing prey or finding mates. The scene usually contains many potential targets, each of which may be weak or ambiguous, so there is strong selective pressure to perform this task efficiently. Doing so requires at least two levels of non-trivial perceptual inference. The first is to accurately estimate the likelihood of the target location so that little time is spent on false targets. The second is to accurately integrate information over time and across actions, so that the new information obtained during prolonged vigilance and after a head or eye movement is updated with the old. This integration of information, past, present, and possibly across modality, contributes to an internal representation of target location in the larger scene. A computational model of such an integrative, inferential memory was developed by Najemnik and Geisler (2005, 2009) for optimal visual search, in which a foveated visual system is used to search for a target in noise. Uncertainty of target location increases with eccentricity due to decrease in ganglion cell density, but each successive saccade provides additional information which the model integrates to compute a likelihood map of target location. In a real biological system, this type of map would not be 2D, but represent the 3D scene and factor feedback from eye, head, and body-movements, and potentially information obtained through other modalities such as the auditory soundscape and movement of the target.

#### Inference of 3D scene structure

Animals act in a 3D world, and the representations needed to guide actions such as navigation or visual search must encode many aspects of 3D scene and target structure. As discussed above, psychophysical research has shown that early in perceptual processing the representations used by humans take into account the 3D scene relationships preferentially over 2D patterns (Nakayama et al., 1995; Lee and Spelke, 2010). This has important implications for internal representations, because it implies that information about the scene is represented and processed in a format that encodes the 3D structure of the environment. This representation need not be exhaustive or detailed, and it need not form a “map” of the 3D scene. We would expect such representations to have complex properties, because they must appropriately factor in movements of the eyes, head, ears, and body, as well as motions of other objects in the environment (Colby and Goldberg, 1999; Melcher, 2011; Tatler and Land, 2011). These representations must also integrate information across multiple sensory modalities to form a common representation of the 3D environment that serves multiple behavioral goals such as foraging, pursuit of prey, communication, locomotion, and navigation.

A particularly challenging aspect of spatial representation is that it must remain coherent despite changes in the raw sensory information that occur due to self-motion or the motion of other components of the scene (Byrne et al., 2007; Melcher and Colby, 2008; Melcher, 2011). The problem the animal faces is that although many components of the scene are stable, such as the shape of the terrain or the positions of obstacles, the sensory input rarely is because the animal and its sensory organs move. Thus, the animal’s actions and behavior must be based on the properties of the scene and not the fluctuating sensory information. The problem is further complicated for dynamic aspects of the scene, such as moving targets, because the representation must also predict trajectories, although this can also provide information that make the target standout from the background.

The nature of the representation of spatial structure (e.g., whether it is referenced to the scene, the body, or past motor actions) remains an active area of research (Burgess, 2008; Tatler and Land, 2011), and it is not clear how many distinct forms of spatial structure are necessary to subserve scene analysis tasks such as target search, pursuit, locomotion, or path planning. However, the general function of higher-level representations is to transform and integrate the lower-level information and feedback from sensorimotor action representations to form a consistent and cumulative representation of the external scene which can then drive behavior. Even in tasks such as auditory scene analysis, the spatial locations of the sound sources can play a role in helping to identify and extract them (Shinn-Cunningham et al., 2007; Chiu et al., 2009). In this sense, the object and spatial memories can be mutually informative – as representations of one type become more fully formed, they help inform the other (as indicated by the double arrow).

#### Actively driven perception

Animals actively probe their environment and take actions based on both their current sensory input as well as on the accumulated information acquired from past actions. This means that the sensory input can change dramatically from instant to instant with the animal’s actions. Perceptual continuity or coherence relies on integration of the new sensory information with the internal representation maintained by the system. Actions could be as simple as a head turn to disambiguate the location of a sound or as complex as a sequence of eye movements during visual search. The choice of action must be carefully selected to rapidly and reliably acquire scene information and progress toward the behavioral goal.

The *behavioral state* node occupies the deepest level in the scene analysis framework (**Figure 6**), and sits intermediate between the sensory and motor areas. On the sensory side, this area coordinates the perceptual processing necessary to achieve specific behavioral goals. There are more direct sensory-motor interactions at lower levels, but a higher-level representation of the behavioral state is needed because the current behavioral goal affects both how the sensory input is processed and the appropriate action to take in response, as well as the sensory inputs to follow. For example, the information that must be extracted from the scene during foraging is very different from that used in mate selection. We use the term state in a broad sense to represent an animal’s current mode of behavior. This could be directed toward a specific goal (e.g., foraging for food, pursuit of a target, mate selection, etc.), and it could also represent intermediate states of the system while it progresses toward a goal, such as speed of locomotion, planning target interception, and so on. The behavioral state must also represent information related to progression toward the goal. For example, in target pursuit, the goal represents the relative spatial position or predicted path of the target; or during foraging, information about the values associated with potential food targets; in mate selection, a wide range of fitness signals must be integrated to drive courtship behavior.

The top-down feedback influences or control the types of signals extracted from the scene in both the target and spatial memories. During visual search, information about the target’s likely form and spatial location is transmitted to lower areas and help locate it more efficiently in a scene. In auditory scene analysis, whether a subject attends to a voice, the music in the background, or the sound of something moving across the floor, all depend on the current task, which determines what kinds of acoustic information are extracted from auditory input. Mate selection is another example, where highly specific information, often across multiple modalities, need to be derived from the scene. These examples imply that either there are multiple parallel circuits in the system specialized for specific tasks, or that the neural circuits are more generic, but highly reconfigurable so that they adapt to a wide range of tasks.

In addition to coordinating the sensory side, the behavioral state also drives action. The action of the system is an integral part of scene analysis behavior, and understanding the resultant motor actions has proven crucial, for example, in echolocating bats, for understanding the sensory representations and computations. In the framework, this is depicted by the path and motor planning node and an additional lower-level node for specific motor actions, responses, and locomotion. Like for sensory processing, the behavioral state also influences the nature of the sensory-motor interaction at lower levels, and these have distinct neural substrates in the form of parallel circuits or a more general circuit with top-down input.

There is a broad range of motor actions that can aid scene analysis. On the shortest time scale, *compensatory actions* facilitate scene analysis by stabilizing the projection of the scene onto the sensor, such as smooth pursuit eye movements or the head bobbing reflex in pigeons. Tracking actions represent movements involved in pursuit or locomotion and are also coordinated dynamically with the ongoing sensory input. These actions are driven directly by representations at lower levels, and can be further guided or modulated using information from the behavioral state, feedback or efference copy. *Probing actions* are the most interesting from the viewpoint of scene analysis because they play a crucial role in solving otherwise insoluble problems. Accurately directed head or eye movements during visual search are one type of action already discussed, which actively probe the scene to efficiently locate the target. Other examples include head and pinnae movements used to disambiguate sound source location or haptic probing with hands or whiskers. Animals that rely on active sensing, e.g., echolocating bats and cetaceans, as well as electrolocating fish, adjust the signals they produce as they probe the environment. Probing actions are also used to aid object discrimination and identification. An actively studied question is to what extent probing actions are ideal in the sense of providing the most information about an object and whether information gathered across multiple movements is integrated to form more accurate spatial representations or increased resolution. More generically, actions are initiated in response to an inference or decision, such as whether an animal is close enough to strike at a target, and advance the animal toward its behavioral goals.

## CONCLUSION

We have presented a framework that attempts to encompass the set of scene analysis problems that are relevant to a wide range of animals, including humans. While most of our classical notions of scene analysis come from studying aspects of human behavior, such as auditory scene segmentation and streaming (Bregman, 1990) or perceptual organization in vision (Palmer, 1999), it is clear from the perspectives presented above that scene analysis covers a much broader range of problems. Furthermore, it forces us to go beyond the laboratory setting and grapple with the issue of how animals and humans process the wide variety of complex, natural stimuli in their natural habitats. The diversity of animal systems and their natural environments provides a wealth of examples from which the most appropriate models can be selected to address specific issues in natural scene analysis.

We selected four animal examples that highlight these different aspects of scene analysis, but there are many other animals and behaviors that also illustrate these principles. For each of the proposed properties discussed above, one can ask to what extent does a given animal require this property for scene analysis? For example, do electric fish use higher level structural knowledge to recognize objects? To what extent do songbirds integrate sounds across time into auditory streams? Answering questions like these will require the development of more sophisticated computational models, a better characterization of the sensory signals in natural scenes, and more detailed studies of animal perception and action in their ecological niche.

A given animal’s perceptual strategy will lie at some point along a continuum between simple signal detection and general purpose scene analysis, and understanding where this point is requires characterizing the limits of an animal’s abilities under a range of task difficulties. For example, a jumping spider detecting a fly against a uniform or blurred background is simpler than detecting it against a complex surface. For a particular task, we might expect that an animal has evolved solutions that approach that of an ideal observer, given the physical constraints and task demands of the system. At some point, however, the difficulty of the task will exceed limits of the system, e.g., how accurately does the songbird recognize song with an increasing number of competing songs? Knowing these limits will inform us about the extent to which an animal performs scene analysis and could provide important insights into how it is carried out. Fundamentally, perceptual performance is constrained by the underlying computations. One of the goals of this paper is to promote computationally guided experimental investigations that will help reveal the underlying scene analysis processes used in different animal systems.

For most animals, and especially human observers, we do not have good computational models for solving scene analysis tasks. We do not know, for example, how to identify objects against a complex background or under occlusion. We have an incomplete understanding of the computations required for auditory scene segregation and recognition in complex acoustic environments. Echolocation and electroreception are even more mysterious. These are not just mysteries about specializations in biology, but highlight questions about the computational principles that enable scene analysis in any system, biological or machine. Although there continues to be progress and even success in restricted domains, these are still many unsolved problems. The difficulties increase when we consider scene analysis problems beyond pattern recognition. For example, what information about the 3D environment is needed to guide locomotion? How is this extracted from the raw sensory signals, and what are efficient ways of doing this? What are the principles that govern the perception–action loop? Research on these questions is still in its early stages, and the models that come out of these efforts will be important for advancing our understanding of the computational problems in scene analysis.

This underscores perhaps the most important point of this article: studies of animal systems, their behavior, environment, and limitations sheds light on *what scene analysis problems need to be solved*. Animals have evolved sensors and information processing systems that are optimized to carry out a repertoire of scene analysis tasks. We cannot directly observe how information is processed by the system because subserving any observable behavior is a myriad of sub-tasks working in concert. Models of those tasks constitute hypotheses about how information is processed in the system, and so the merit of a model is determined by the extent to which it explains and predicts aspects of animal behavior. Thus, uncovering the essential computations in scene analysis is a *scientific* process. This stands in contrast to engineering approaches where algorithm development is guided by performance on tasks that are well-defined, but often fail to capture the robustness and adaptability of animal systems. Furthermore, even robust and well-defined computational algorithms do not necessarily have ecological relevance. For example, auditory stream segregation is often defined with the goal of recovering the individual waveforms of the different sound sources, but this is not necessarily the problem animals need to solve. Comparisons between the computational models and biological systems are necessary to guide further development and provide a means to identify models that are the most relevant.

Our goal in this article is to expand the concept of scene analysis to consider how both humans and animals perceive and interact with their natural environment. In contrast to psychophysical approaches that focus on humans and carefully controlled stimuli, we emphasize the need to study how a wide range of animals deal with the complex sensory signals that arise from natural behavior in the real world. In contrast to engineering approaches to specific scene analysis problems such as object recognition or speech recognition, here we have emphasized the need for models that have potential ecological relevance and can guide experiments and inform the interpretation of data. Behavioral and physiological studies can only go so far without detailed computational models of the information processing. Recording an animal’s sensory environment and its actions is not sufficient to gain insight to the computations underlying its behavior because the range of environmental variables and behavioral repertoires is too large to be measured exhaustively. Models of the information processing guide us in how to pare down or prioritize the essential dimensions of this space. Our goal here has been to better define what information processing is needed to solve the scene analysis problems faced by both humans and animals. Viewing scene analysis from this broader perspective we believe holds the greatest promise for elucidating how it is solved throughout the animal kingdom.

## AUTHOR CONTRIBUTIONS

All authors contributed equally to this work.

## Conflict of Interest Statement

The authors declare that the research was conducted in the absence of any commercial or financial relationships that could be construed as a potential conflict of interest.
