# Corrigendum: Scene analysis in the natural environment

**DOI:** 10.3389/fpsyg.2014.01119

**Published:** 2014-10-09

**Authors:** Michael S. Lewicki

**Affiliations:** Electrical Engineering and Computer Science Department, Case Western Reserve UniversityCleveland, OH, USA

**Keywords:** active perception, auditory streaming, echolocation, vision, electroreception, scene analysis, neuroethology, top-down processes

In the original article, the caption of Figure 5 contained an error of acknowledgment for the figure source. It should be: (redrawn from von der Emde, 1999).

## Conflict of interest statement

The author declares that the research was conducted in the absence of any commercial or financial relationships that could be construed as a potential conflict of interest.
